# Risk of recurrent stillbirth and neonatal mortality: mother-specific random effects analysis using longitudinal panel data from Indonesia (2000 – 2014)

**DOI:** 10.1186/s12884-022-04819-4

**Published:** 2022-06-28

**Authors:** Alka Dev

**Affiliations:** The Dartmouth Institute for Health Policy and Clinical Practice, Geisel School of Medicine at Dartmouth College, Lebanon, NH USA

**Keywords:** Stillbirth, Neonatal mortality, Longitudinal risk, Adverse birth outcomes, Indonesia, Random effects analysis, Birth history

## Abstract

**Background:**

Despite significant government investments to improve birth outcomes in low and middle-income countries over the past several decades, stillbirth and neonatal mortality continue to be persistent public health problems. While they are different outcomes, there is little evidence regarding their shared and unique population-level risk factors over a mother’s reproductive lifespan. Data gaps and measurement challenges have left several areas in this field unexplored, especially assessing the risk of stillbirth or neonatal mortality over successive pregnancies to the same woman. This study aimed to assess the risk of stillbirth and neonatal mortality in Indonesia during 2000–2014, using maternal birth histories from the Indonesia Family Life Survey panel data.

**Methods:**

Data from three panels were combined to create right-censored birth histories. There were 5,002 unique multiparous mothers with at least two singleton births in the sample. They reported 12,761 total births and 12,507 live births. Random effects (RE) models, which address the dependency of variance in births to the same mother, were fitted assuming births to the same mother shared unobserved risk factors unique to the mother.

**Results:**

The main finding was that there having had a stillbirth increased the odds of another stillbirth nearly seven-fold and that of subsequent neonatal mortality by over two-fold. Having had a neonatal death was not associated with a future neonatal death. Mothers who were not educated and nullipara were much more likely to experience a neonatal death while mothers who had a prior neonatal death had no risk of another neonatal death due to unmeasured factors unique to the mother.

**Conclusions:**

The results suggest that for stillbirths, maternal heterogeneity, as explained by a prior stillbirth, could capture underlying pathology while the relationship between observed risk factors and neonatal mortality could be much more dependent on context. Establishing previous adverse outcomes such as neonatal deaths and stillbirth could help identify high-risk pregnancies during prenatal care, inform interventions, and improve health policy.

## Background

Nearly half of the estimated 5.2 million under-five deaths occurring worldwide in 2019 were among neonates [1]. A high neonatal mortality rate reflects antetnatal, intrapartum, and neonatal care gaps and serves as a measure of the health system’s capacity to save newborn lives [2]. Poor maternal and newborn healthcare quality also contributes to the stillbirth rate, with equally profound social, psychological, and economic consequences for families and healthcare workers [3–5]. Pregnancy risk factors such as poor micronutrient intake, low maternal body mass index (BMI), poor quality of antenatal care, and low access to emergency obstetric services during labor and delivery are shared causes of stillbirths and neonatal death [6]. Further, undocumented complications during labor and delivery may contribute to a third of stillbirth and neonatal mortality burden in low-income countries [7]. Previous research has found that these deaths and their consequences could be concentrated in certain families/mothers and communities. In India, for example, Das Gupta found recurrence of child deaths in a few families in 11 villages in Punjab where 12.6% of mothers accounted for 62.2% of child deaths [8]. In Brazil, Sastry found significant clustering of mortality risk among siblings in the Northeast where 60% of the deaths occurred in 9% of families [9].

More recent survey data from India also supports the clustering of neonatal mortality, with a twofold increase in the odds of subsequent neonatal mortality among mothers who have had a prior neonatal death [10]. In Tanzania, investigators found that a woman who lost her first baby due to stillbirth had five times the risk of losing her next pregnancy to stillbirth and twice the risk of having an early neonatal death compared to a woman who had not had a stillbirth [11]. Similar associations were found in a facility-based survey from 24 countries in Africa, Asia, and Latin America [12]. After adjustments for small size for gestational age and preterm status, the odds of having a stillbirth or neonatal death in a second pregnancy were twice as high for women who had a prior stillbirth than for women who did not have a previous stillbirth. In a pooled meta-analysis of 16 published studies, Lamont and colleagues found that even in high-income countries, women who had a stillbirth in their first pregnancy were almost five times more likely to experience a stillbirth in their second pregnancy [13].

This evidence suggests that some births may not be genuinely independent observations, as certain women may have been more likely to experience an adverse birth outcome due to inherent risk factors. These *mother-specific risk factors*, i.e., risk factors attributable to the mother that yield different risk profiles across different mothers, may impact consecutive births but are unobserved or unaccounted for in the available data. Vaupel referred to this dependence as unobserved heterogeneity or ‘frailty’ (referred to as vulnerability in this paper) and showed that it could bias the interpretation of cohort and period mortality rates for individuals who have different (but constant) underlying overlooked risks affecting mortality [14]. The concept of unobserved heterogeneity has also been applied in public health research for meta-analysis (clustering within studies) [15], randomized trials (clustering within study sites) [16], hospital or school-based studies (clustering by sampled sites) [17], and neighborhood-level studies (clustering by neighborhood) [18]. Analyzing the clustering of stillbirth and neonatal deaths among mothers in low and middle-income countries could add valuable evidence to our understanding of risk assessment and maternal health monitoring but is limited by the paucity of longitudinal birth history data in these settings. This study aimed to address this gap by evaluating the risk of stillbirth and neonatal mortality in Indonesia during 2000–2014, using longitudinal maternal birth histories from the Indonesia Family Life Survey (IFLS) panel data.

## Methods

This study used the IFLS, a longitudinal household survey, to estimate the contribution of mothers’ observed and unobserved characteristics to their risk of stillbirths and neonatal mortality. This study is innovative because it accounts for statistical dependence between births to the same mother using complete pregnancy histories from household survey data, allowing for evaluations of stillbirth and neonatal mortality in the same population.

### Data

IFLS is a population-representative, longitudinal survey of 13 (of 27) provinces consisting of five panels: 1993, 1998, 2000, 2007–08, and 2014–2015. In 1993, IFLS covered an initial sample of 7,224 households spread across six islands. This sample was selected to maximize the representation of Indonesia's cultural and socioeconomic diversity, representing approximately 83% of the Indonesian population at the time, while being cost-effective [19]. Three hundred twenty-one enumeration areas (EAs) or clusters were chosen from an existing sampling frame of about 60,000 households. Urban clusters and those in smaller provinces were over-sampled to facilitate urban–rural and Javanese to non-Javanese comparisons. Field teams randomly selected 20 households in each urban cluster and 30 households in each rural cluster for inclusion. In the subsequent panels, the goal was to relocate and re-interview all households interviewed in the previous panels, including people that moved to another IFLS province [20]. Individuals that split off from the original IFLS household but remained in the 13 provinces were also interviewed but given new household IDs.

IFLS included a household survey, with adult and children’s questionnaires, as well as a community and facility survey. The adult questionnaires included modules on education, marriage, migration, employment, health status, utilization of health services, individual and household assets, fertility and contraception, infant feeding practices, as well as proxy data on household members who were away. All ever-married women were asked questions about marriage, contraceptive use, pregnancies and outcomes, use of antenatal care, children ever born, infant feeding, and the status of child survival.

### Sample Selection

Due to missing date and gestation data, especially for stillbirths, birth histories reported in 1993 and 1998 were excluded. The newer datasets were much less likely to be missing gestational age for each birth, which was important for defining stillbirths, as well as the year of birth which was important for removing duplicates across panels. Births reported in the 2000, 2007, and 2014 panels were extracted to produce birth histories for mothers for births ending in stillbirth or live birth. All self-reported miscarriages were deleted, but miscarriages with gestation over 7 months were recoded as stillbirths. Similarly, self-reported stillbirths with gestation under 7 months were recoded as miscarriages and excluded. Duplicates reported in subsequent panels were removed based on year of birth and outcome. Births that were missing gestation and other covariates were excluded (l.0%). For the analysis, only multiparous women were included in the final sample. Data were right-censored since some mothers had not completed their reproductive span at the time of the final survey. The data were also panel unbalanced, meaning that mothers had different numbers of births in the sample.

### Primary risk factors

Birth histories of mothers were used to calculate two primary risk variables for each birth: any history of stillbirth and any history of neonatal death. For stillbirth history, a binary variable was constructed with a value of 1 if the mother had a prior stillbirth and 0 if not. For neonatal mortality history, a binary variable was constructed with a value of 1 if the mother had a prior live birth that resulted in neonatal death and 0 if not.

### Outcomes

There were two outcomes evaluated in separate models: stillbirth, defined as a pregnancy with seven months (or 28 weeks) or longer gestation that ended in a fetal death, and neonatal mortality, defined as death in the first 28 days of life per World Health Organization (WHO) recommendations [21]. For neonatal mortality, a one-month endpoint was used to capture deaths that were reported in months, which was a majority of the sample. Gestational age was self-reported by the woman during the survey.

### Covariates

Covariates were measured at the level of birth, mother, and household. For example, pregnancy duration and parity were specific to birth, education was specific to the mother, and urban residence was specific to the entire household.

#### Number of births to date

Calculated as the number of births (live and still) that a woman reported across the full birth history. Each birth was assigned a value based on this history, grouped into three categories: none, 1–2 birth, and 3 or more births.

#### Age of mother at birth

The age of a mother at birth was calculated as the difference in years between her birth year and the year of each one of her births. The age of the mother at the time of the survey was not used in the analysis. Any ages below 15 years were excluded.

#### Education

Education level was measured at the time of the survey. It was introduced as a categorical variable in the following four groups: none, elementary, junior high, senior high, college, or religious/vocational. ‘Religious’ education included *Madrasah* Islamic schools.

#### Urban–Rural

Whether a woman lived in an urban or rural cluster was based on her IFLS cluster, which was listed as urban or rural in the sampling frame [22].

Additional covariates that would have been useful but excluded because they were missing data for a significant proportion of women included access to antenatal care, place of birth, and skilled care at birth. Data on maternal body mass index (BMI) and household per capita expenditures were also available in the survey but only measured at the time of the interview. For many births, these would be far removed from the year of birth and were not used to avoid spurious correlations.

### Analytical Approach

This study uses Random Effects (RE) models to estimate the underlying unmeasured subject/mother-specific risk of having either a stillbirth or a neonatal death, given observed group-level risk factors and covariates among women with at least two births in the sample. RE models describe variation in mother-specific responses according to unmeasured characteristics. Using the RE approach allows the mother-birth relationship to have mother-specific effects assuming this relationship varies by mother but is similar for births to the same mother. In RE models, all unmeasured factors are assumed to contribute to heterogeneity regardless of whether they can ever be observed. This variance is allowed to be random and uncorrelated between mothers and observed variables and allows for the estimation of the effect of maternal independent variables that remain constant across births (such as education and urban/rural residence) regardless of the number of observations in each cluster. The combined variance of unobserved heterogeneity is output as *rho* in the model statistics [23]. It can be interpreted as the variation in the odds of having a stillbirth (or neonatal death in a neonatal model) that was not explained by the observed variables in the model.

### Model building and standard error adjustment

The model-building strategy was based on the evaluation of biological and socioeconomic factors from relevant public health literature. Biological factors included age of the mother at birth, parity, female birth (neonatal model only), and any prior history of a stillbirth or neonatal death. Socioeconomic factors included urban or rural residence and education level. Some independent variables' exposure timing can produce causal estimates, while others can only be interpreted as correlates. In particular, the age of the mother at birth, parity, and history of stillbirth or neonatal death could be established as occurring before birth. Therefore, estimates for these variables can be interpreted as predictive of the outcome. Mothers’ education level and urban residence could only be correlated with the outcome as they were measured at the time of interview. However, they could not be evaluated as predictive or causal as it was not possible to determine whether these characteristics were true at the time of birth. Male birth would be associative but not necessarily causal in the absence of other information but established research has shown higher mortality among male newborns [24].

Standard errors were adjusted for robust estimates for maternal clustering. Clustering within communities was not adjusted to avoid adding greater complexity to an already complex dataset and survey design. Shared community-level factors that impact stillbirth and neonatal mortality that may exist would be included in maternal clustering estimates, introducing bias in interpreting maternal effects. Hierarchical clustering affects standard errors but does not influence effect estimates. All analyses were performed in Stata statistical software version 17 [25]. Statistical significance was set at 0.05 and *p-*values were 2-tailed. Calculations were weighted to account for survey design and attrition over time and to adjust estimates for the Indonesian population in the panel year.

## Results

### Sample description

There were 5,002 unique multiparous mothers reporting at least two singleton births, of whom 1,303 were new in the 2000 panel, 2,461 were new in the 2007 panel, and 1,238 were new in the 2014 panel. Approximately 69.2% (*n* = 902) of mothers interviewed in 2000 were also interviewed in 2007 and 23.2% (*n* = 302) were also interviewed in 2014. An additional 11.6% (*n* = 151) were interviewed in the 2000 and 2014 panels but not in 2007. Table 1 presents sample means and percentages of demographic characteristics by panel. A key difference of note is the higher mean age of respondents over panels which corresponds to much greater proportions of women over 35 in 2014 compared to 2000 (45.2% versus 15.9%). Mean parity over this time decreased from 2.0 births per woman to 1.6 births. The proportion of women who had more than 2 births also decreased (from 66.6% in 2000 to 45.4% in 2014).

**Table 1:** Demographic characteristics and outcomes for mothers by panel year

	**2000**	**2007**	**2014**
Number of mothers	1,303	3,363	3,438
Number of total births	2,071	5,165	5,525
Number of live births	2,040	5,067	5,400
**Weighted sample means (standard deviation)**			
Mean age	26.2 (5.5)	28.5 (5.6)	32.9 (5.2)
Mean parity	2.0 (1.2)	1.7 (1.0)	1.6 (0.9)
**Weighted sample proportions (%)**			
Age Group (years)			
15–19	6.9	2.7	< 1
20–24	21.6	17.8	5.9
25–29	30.0	32.0	21.9
30–34	25.6	29.4	36.9
35 +	15.9	18.1	35.2
Parity Group			
2	33.5	48.8	54.7
3–4	49.5	40.8	36.5
5 +	17.1	10.4	8.8
Education level			
None / Primary	44.6	32.4	25.9
Junior High	14.4	18.1	19.7
Senior High	24.5	26.4	28.7
College	7.9	11.8	15.1
Religious/Vocational	8.6	11.3	10.6
Setting			
Urban	45.3	48.3	50.6
Rural	54.7	51.7	49.4
Female births (live births only)	47.5	47.7	48.8
**Weighted rates**			
Stillbirth Rate (per 1,000 total births)	13.4	18.8	24.9
Neonatal Mortality Rate (per 1,000 live births)	23.1	16.3	17.7

The proportion of women with no formal education or only primary education decreased across the three panels, from 44.6% in 2000 to 25.9% in 2014. Consequently, the proportion of women with a college education nearly doubled from 8.6% in 2000 to 15.1% in 2014. There was a 23.2% increase in the proportion of women attending religious or vocational school between 2000 and 2014 the majority of women in this category attended religious schools. There were proportionally more urban mothers in the 2015 panel than in the 2000 panel. A little less than half of all live births were girls, and there were no meaningful differences in this proportion over each panel. The stillbirth rate increased from 13.4 fetal deaths per 1,000 total births in the 2000 panel to 24.9 in the 2014 panel, while the neonatal mortality rate decreased from 23.1 neonatal deaths per 1,000 live births in the 2000 panel to 17.7 neonatal deaths in the 2014 panel.

Random Effect Models.

Table 2 and Table 3 show results from fixing RE models for the odds of having a stillbirth or neonatal death, respectively, given observed independent variables. The following section describes each outcome’s results and statistical measures for goodness-of-fit. Table 2 shows mother-specific odds ratios for having a stillbirth given the history of stillbirth, parity, residence, education, and age at birth predictors with a dummy variable for panel year to adjust for differences in panel-specific factors. Interpretations for the full model are presented here.

**Table 2:** Adjusted odds ratios of selected risk factors for stillbirth in Indonesia (2000 – 2014)

Variables	Null model	Intermediate Model	Full model
*History*			
No prior stillbirth			*ref*
Prior stillbirth			6.46^***^
*Number of prior births (parity)*			
None			1.52^*^
One or two			*ref*
Three or more			0.81
*Residence*			
Rural		*ref*	*ref*
Urban		1.11	1.10
*Education*			
No schooling		1.90	1.56
Elementary		*ref*	*ref*
Jr. High		1.24	1.17
Sr. High		1.06	1.02
College		0.69	0.66
Religious/Vocational		2.02^**^	1.85^**^
*Age of the mother*			
Age-squared		1.01^***^	1.00^**^
Age		0.72^***^	0.78^**^
*Panel year*			
2000	*ref*	*ref*	*ref*
2007	1.28	1.24	1.30
2014	1.50	1.64^*^	1.70^**^
Constant	0.005	0.33	0.21
Number of births	12,761	12,761	12,761
Number of mothers	5,002	5,002	5,002
Log likelihood	-1218	-1203	-1199
AIC	2444	2430	2427
BIC	2474	2520	2539
*rho*	0.42	0.41	0.0015

^***^p ≤ 0.001, ^**^p ≤ 0.01, ^*^p ≤ 0.05

**Table 3:** Adjusted odds ratios of selected risk factors for neonatal mortality in Indonesia (2000 – 2014)

VARIABLES	Null model	Intermediate model	Final model neonatal death history	Final model stillbirth history
*History*				
No history			*ref*	*ref*
Prior neonatal death			1.37	
Prior stillbirth				2.59*
*Sex of newborn*				
Male			*ref*	*ref*
Female			0.59^***^	0.59^***^
*Number of prior births (parity)*				
None			1.99^**^	2.06^***^
One or two			*ref*	*ref*
Three or more			1.26	1.10
*Residence*				
Rural		*ref*	*ref*	*ref*
Urban		0.74	0.72^*^	0.71^*^
*Education*				
No school		2.85^*^	2.87^*^	2.94^*^
Elementary		*ref*	*ref*	*ref*
Jr. High		1.19	1.13	1.11
Sr. High		0.80	0.73	0.72
College		0.70	0.60	0.58
Religious/vocational		0.81	0.75	0.73
*Age of the mother*				
Age-squared		1.00^**^	1.00	1.00
Age		0.75^**^	0.83	0.83
*Panel year*				
2000	*ref*	*ref*	*ref*	*ref*
2007	0.73	0.69	0.77	0.77
2014	0.71	0.86	1.05	1.04
Constant	0.0073^***^	0.072	0.13	0.11
Births	12,506	12,506	12,506	12,506
Number of mothers	5,002	5,002	5,002	5,002
Log likelihood	-1093	-1073	-1059	-1057
AIC	2194	2169	2151	2145
BIC	2224	2258	2270	2264
*rho*	0.37	0.35	0.31	0.36

^***^p ≤ 0.001, ^**^p ≤ 0.01, ^*^p ≤ 0.05

A woman who had a prior stillbirth had a 6.5-fold higher risk of having another stillbirth than if that woman had not had a prior stillbirth, all else being equal. A woman was also 1.5 times more likely to experience a stillbirth in her first birth than in subsequent births. Whether the mother lived in an urban area versus a rural one was not associated with having a stillbirth, all else being equal for that mother. The effect of education was insignificant for all levels except a mother who attended a religious or vocational school; she had nearly twice the odds of having a stillbirth than if she had received an elementary education only. The effect of age became slightly more pronounced as a woman aged, given her set of observed characteristics. Categorical age variables remained insignificant at all ages (results not shown).

The final model showed an improvement in the log-likelihood over the null model (χ^2^(13) = 43.01, *p* = 0.0000). RE models can also be assessed based on the rho or intraclass correlation coefficient, representing the correlation between the (latent) risk of having a stillbirth across two births for the same mother. For the intermediate model, the *rho* was 0.41 meaning that 41% of the variance of the risk of stillbirth, beyond that explained by age, residence, education, and panel, could be attributed to other characteristics of the mothers. For the full model in Table 2, the *rho* was 0.0015, meaning that after adjusting for prior stillbirth, less than 1% of the latent risk of having a stillbirth was attributed to unobserved characteristics of the mother. Thus, prior stillbirth history was a key component of a mother’s underlying vulnerability. Another way of thinking about this is that around 41% of her vulnerability in successive births was explained by risk factors that led to her prior stillbirth and the experience of the stillbirth itself, after adjusting for her parity, residence, education, and age at birth.

Table 3 shows mother-specific odds ratios for having a neonatal death given prior history, parity, residence, education, sex of the child, and age at birth predictors with a dummy variable for panel year to adjust for differences in panel-specific factors. Interpretations for the full model are presented here. Having a prior neonatal death did not affect the odds of having another neonatal death for any mother, all else being equal. However, having had a prior stillbirth increased the odds of having a future neonatal death by a factor of 2.59. Having a female newborn was protective against neonatal mortality, as girl babies were 41% less likely to die than boy babies with the same mother. A woman was twice as likely to experience a neonatal death in her first birth than in subsequent births. A mother had 29% lower odds of having had a neonatal death if she lived in an urban area than if the same mother had lived in a rural area, all else being equal for her. The effect of education was significant for no education; a mother who had no education had nearly three times the odds of having a neonatal death than if she had received an elementary education. There was no effect of maternal age at birth on neonatal mortality. There were no panel-specific effects on neonatal mortality.

The final models showed an improvement in the log-likelihood over the null model for neonatal death history (χ^2^(12) = 67.66, *p* = 0.0000) and for stillbirth history (χ^2^(12) = 73.32, *p* = 0.0000). For the full models in Table 3, the *rho* was 0.31 with adjustment for prior neonatal death, meaning that 31% of the latent risk of having a neonatal death was attributed to other unobserved characteristics of the mother. After adjusting for stillbirth history, the *rho* was 0.36 as in the intermediate and null models, suggesting that given prior stillbirth, another 36% of the latent of having a neonatal death was attributed to other unobserved maternal characteristics. Thus, for a mother, prior stillbirth history did not explain additional underlying vulnerability for neonatal death than the intermediate models, as it had for an additional stillbirth. Neonatal death was still vulnerable to unobserved maternal as was not the case for stillbirths, after adjusting for neonatal death or stillbirth history, sex of the newborn, parity, residence, education, and maternal age at birth.

## Discussion

This study estimated the effects of selected independent variables on the odds of a pregnancy resulting in stillbirth or neonatal death conditional on the maternal risk profile for women interviewed in Indonesia between 2000 and 2014. The main finding was that having one stillbirth increased the risk of having another. Similar trends have been observed in other countries. Compared to the seven-fold increase in the odds of stillbirth in our study, in Finland having a stillbirth increased the risk of future stillbirths and spontaneous abortions by 20% even after controlling for all major obstetric complications [26]. In a pooled analysis of births from Finland, Malta, and Scotland, women with stillbirth in the first pregnancy were twice as likely to have a subsequent stillbirth [27] and similar results were reported in the Netherlands as well [28]. Using random effects to adjust for unobserved heterogeneity or maternal vulnerability, we also found that the risk of having recurrent stillbirth could be attributed to observed maternal characteristics, which included the prior stillbirth. Therefore, the first stillbirth was a good approximation of underlying vulnerability for subsequent stillbirths, which may be biological, contextual, or a combination of several factors. Given that similar associations are observed in high-income countries as noted above, it is possible that the unobserved heterogeneity or maternal vulnerability for the risk of recurrent stillbirth is not only driven by access to and the quality of maternal health services.

The main finding regarding neonatal deaths was that after adjusting for unobserved heterogeneity, having had a prior neonatal death was not predictive of neonatal death in future live births, but having had a prior stillbirth increased the odds of having a future neonatal death. However, contrary to the findings in our study, previous history of neonatal mortality was also associated with future risk for the same in India [10] and other low- and middle-income countries [29]. The risk of a neonatal death across births could not be estimated to the same degree by the observed variables as the risk of stillbirths; the risk of having a neonatal death was not entirely due to maternal factors since nearly one-third of the variation in risk of neonatal mortality in this sample could not be attributed to the mother. In Bangladesh, a second neonatal death was associated with a history of neonatal mortality when the causes of death had been non-infectious diseases, suggesting an intrinsic and shared maternal risk whereas for a first neonatal death due to an infection, there was no association with subsequent neonatal death, suggesting that parents can avoid a preventable hazard, if known [30].

Given that the role of prior stillbirth and neonatal mortality was starkly different across pregnancies, a prior stillbirth may capture ongoing maternal risk factors whereas a prior neonatal death could have risk factors not shared across births to the same mother. These could be attributable to unobserved baby-level factors or maternal and neonatal health service factors. Several variables were missing data in the sample for neonatal mortality, which could have helped resolve the magnitude of unobserved heterogeneity. These included measures of use of prenatal services, pregnancy complications and maternal morbidity, the place of birth, the quality of labor and delivery care, birth weight, neonatal care, and breastfeeding. In high-income countries, there is a very low risk of recurrent neonatal death given the quality of services, A stillbirth followed by a neonatal death, as found in this study, could be indicative of the underlying maternal risk for adverse birth outcomes shared across pregnancies.

As expected, living in an urban area was correlated with lower neonatal mortality risk but not associated with stillbirth. This finding supports the notion that stillbirths may have risk factors that are not addressed sufficiently in poorly resourced health systems, although some risk seems to be carried out due to maternal genetics or pathophysiology that are not addressed even in high-quality care. Attending a religious or vocational school was associated with a higher risk of having a stillbirth than having even an elementary school education. Most of the women in this category had attended a religious school and it is possible that being enrolled in a religious school approximates other cultural beliefs and practices that may carry a higher risk of stillbirth. One potential explanation for this might be consanguinity which is more common in Muslim nations but data on the prevalence of such marriages is scant in Indonesia and assumptions should not be made without further evidence [31, 32]. For neonatal mortality, a mother with no education was more likely to experience a neonatal death than if she had even been to elementary school. Low levels of education in Brazil have been associated with no gains in lower neonatal mortality, compared to intermediate and higher levels of education among mothers, suggesting that women with no or low levels of education do not benefit from neonatal mortality reducing strategies to the same degree [33].

Advanced maternal age was not a substantial risk factor for stillbirths in this study but being younger was associated with a lower risk of stillbirths. Others have found that stillbirth and neonatal mortality risk increases with increasing maternal age even when accounting for maternal morbidities [34, 35]. In low-income countries, in particular, being under 20 or over 35 years has been associated with a higher risk of having a stillbirth [36, 37]. Contrary to other publications, we also did not find any significant contribution of maternal age to the risk of neonatal mortality. One reason for this discrepancy could be poor data quality about self-reported age in population surveys in the absence of vital statistic records, especially among older adults [38, 39]. Similar to published research, this study found that being nulliparous was associated with a higher risk of stillbirth and neonatal death. Data from high and low-income countries show that women who are giving birth for the first time experienced increased odds of stillbirth compared to women with one prior birth [36, 40, 41]. Among younger nullipara women, the risk of having a neonatal death is also higher than those with higher parity (and age) [42] although having more births did not introduce a greater risk of either outcome in our study. Age.

The most critical assumption in this study was that stillbirth and neonatal deaths were not misclassified and therefore, the estimates were valid for these specific outcomes. Misclassification of these outcomes is an important and well-documented issue that could not be resolved here [43]. A second assumption was that recall of birth dates when provided, was accurate, but recall bias has been documented as a significant problem with long-term histories. If incorrect, then the ordering of successive pregnancies could be incorrect, introducing bias in the findings regarding prior history. However, since the findings were in line with published research for this variable, we can assume that the accuracy of these dates was not prone to greater error than dates in other surveys. Finally, for the subject-specific approach, it was assumed that clustering within mothers was more indicative of unobserved heterogeneity than clustering within primary sampling units. It is possible that unobserved heterogeneity also included community-level factors that were attributed to mothers in this analysis. Some of these factors could be geographic.

### Strengths and Limitations

This study has several strengths. First, it uses a novel approach to accommodate clustering of births to the same mother when longitudinal data allows for the analysis of longer birth histories. This approach allows one to investigate the effect of having had a history of adverse outcomes, which is rarely possible from population survey data in low and middle-income countries. Second, using RE analysis, we can model two outcomes that share similar risk factors but show that critical differences in unobserved maternal or community factors, such as health service access and use, impact neonatal survival differently from stillbirths. These community factors are essential considerations in deciding where to invest resources. Because of these potential community factors and greater need for health services, establishing the risk of another stillbirth or neonatal death for a woman who has had a prior stillbirth is important. Targeting antenatal and obstetric services to accommodate both known risks and the potential vulnerability of the fetus or neonate to succumb to unobserved risks, also shines a light on the need for evaluating these two outcomes separately in greater depth.

There are also several limitations. First, there was incomplete data on measures for use of antenatal care, place of birth, and access to newborn care – all factors which can impact birth outcomes. Therefore, the contribution of health system factors was limited and some of these factors could bias the estimation of maternal risk from observed variables. Second, it was not possible to build a birth history over the length of the panels due to missing year of birth data, especially for stillbirths and neonatal deaths in the first two panels. This missing data would have disproportionally biased the results and made it difficult to calculate the mother's age at the time of birth for adverse outcomes. Women with missing data likely had other characteristics that increased their risks such as lesser education, rural residence, older age, or higher parity. Third, it was not possible to adjust for the first stillbirth, which would have no available prior history. In this analysis, the first stillbirth was analyzed as having the same risk as birth with no prior history, which is a flawed assumption. Adjusting for parity was a potential solution and this explained some of the higher odds for nulliparous women. Nonetheless, it would be useful to understand whether there is greater unmeasured vulnerability for first stillbirths. For example, not using health services could be an unexplained vulnerability in first stillbirths.

## Conclusion

Birth history data from population-based surveys have become increasingly important for estimating the global burden of stillbirths and neonatal mortality. The retrospective collection of birth histories results in documentation of multiple births for each respondent. In the analysis of population-based birth history data, the clustering of births within a mother is a concern because birth outcomes are likely to be more similar than different for valuable indicators such as birth weight or gestation. This study improved upon birth-average estimates of risk factors that predict poor stillbirth and neonatal outcomes by adjusting for the effect of shared maternal vulnerabilities across births. The study has implications for collecting birth histories to predict stillbirth and neonatal mortality and suggests that survey-based data collection for these outcomes requires different sets of questions and assumptions. The focus of current household surveys on livebirth histories limits the study of stillbirths. Including both outcomes in the same survey improves the ability to study clustered effects for each. This study supports the policy recommendation that in addition to neonatal mortality, research should focus on establishing the history and potential cause of any stillbirth among pregnant women during prenatal care.

## Data Availability

The data that support the findings of this study are available from RAND Corp. at [https://www.rand.org/well-being/social-and-behavioral-policy/data/FLS/IFLS.html]. They can be requested from RAND Corp with registration at no cost.
